# Soft substrate maintains proliferative and adipogenic differentiation potential of human mesenchymal stem cells on long-term expansion by delaying senescence

**DOI:** 10.1242/bio.039453

**Published:** 2019-04-25

**Authors:** Sanjay Kumar Kureel, Pankaj Mogha, Akshada Khadpekar, Vardhman Kumar, Rohit Joshi, Siddhartha Das, Jayesh Bellare, Abhijit Majumder

**Affiliations:** Department of Chemical Engineering, Indian Institute of Technology Bombay (IITB), Mumbai 400076, India

**Keywords:** Cell mechanics, Mesenchymal stem cells, Senescence, Stemness, Substrate rigidity

## Abstract

Human mesenchymal stem cells (hMSCs), during *in vitro* expansion, gradually lose their distinct spindle morphology, self-renewal ability, multi-lineage differentiation potential and enter replicative senescence. This loss of cellular function is a major roadblock for clinical applications which demand cells in large numbers. Here, we demonstrate a novel role of substrate stiffness in the maintenance of hMSCs over long-term expansion. When serially passaged for 45 days from passage 3 to passage 18 on polyacrylamide gel of Young's modulus *E*=5 kPa, hMSCs maintained their proliferation rate and showed nine times higher population doubling in comparison to their counterparts cultured on plastic Petri-plates. They did not express markers of senescence, maintained their morphology and other mechanical properties such as cell stiffness and cellular traction, and were significantly superior in adipogenic differentiation potential. These results were demonstrated in hMSCs from two different sources, umbilical cord and bone marrow. In summary, our result shows that a soft gel is a suitable substrate to maintain the stemness of mesenchymal stem cells. As preparation of polyacrylamide gel is a well-established, and well-standardized protocol, we propose that this novel system of cell expansion will be useful in therapeutic and research applications of hMSCs.

## Abstract (precis)

**Summary:** hMSCs retain their stemness by delaying senescence when expanded *in vitro* on soft polyacrilamide gel.

## INTRODUCTION

Human mesenchymal stem cells (hMSCs), due to their multi-lineage differentiation potential, immuno-suppressive capability, and immuno-modulatory effects have been used with varying degree of success to treat cardiovascular, musculoskeletal, immune-related and hemopoietic diseases (Pittenger et al., 1999; Ranganath et al., 2012; Tuan, 2013; Wang et al., 2016). Though MSCs are available from multiple adult tissues (Caplan and Bruder, 2001), critically low availability of MSCs in the isolated sample is a major roadblock for clinical trials. For example, in bone marrow aspirates, only 0.001–0.01% of the nucleated cells are MSCs, while a dose of roughly about 100 million cells are required to treat a person 70 kg in weight (Ren et al., 2012). As a result, a long-term *in vitro* expansion is essential to reach a significant number of cells for autologous treatment.

However, on *in vitro* expansion, MSCs lose their proliferative ability and multilineage differentiation potential (Banfi et al., 2000; Wagner et al., 2008). Like any other primary somatic cells, after a certain number of cell divisions, they enter a senescence state, which is morphologically characterized by enhanced spreading area and shape irregularity (Bonab et al., 2006; Wagner et al., 2010b). More importantly, they lose their multilineage potential, migration and homing ability (De Becker and Van Riet, 2016; Honczarenko et al., 2006), making them unsuitable for clinical use (Kassem, 2006; Ullah et al., 2015). Though multiple approaches have been tried to maintain MSC stemness over prolonged expansion (Saei Arezoumand et al., 2017), finding an easy-to-use culture system to achieve the same is still an unmet need. In this context, it might be noted that the NIH on their website has listed six points that need to be addressed to realize the potential of stem cell-based therapies. The first one in that list is “Stem cells must be reproducibly made to proliferate extensively and generate sufficient quantities of cells for making tissue” (Stem Cell Basics IV. | stemcells.nih.gov, 2017, https://stemcells.nih.gov/info/basics/1.htm). A culture system that can fulfill this need may help to progress regenerative medicine significantly.

Controlling the physical microenvironment of the cell culture system might offer a solution in this context. In the past 15 years, it has been shown that mechanical cues such as stiffness of cell culture substrate, shear stress, mechanical strain, cell morphology, substrate topology, etc., influence a wide array of cell behavior and cell fate including survival, proliferation and differentiation (Anderson et al., 2016; Engler et al., 2006; Gilbert et al., 2010; Lutolf et al., 2009; Murphy et al., 2014; Winer et al., 2009; Yeung et al., 2005). It has also been shown that such mechanical cues may play an important role in maintaining MSCs stemness. For example, MSCs cultured on micro-contact printed islands as spheroids and on nano-patterns were shown to retain multipotency and proliferative capacity (Cesarz and Tamama, 2016; Lee et al., 2015; McMurray et al., 2011; Zhang and Kilian, 2013). However, both micro-contact printing and spheroid culture restrict the proliferation of MSCs leading to limited or no expansion in cell number. Moreover, creating micro-patterns or nano-patterns for a large area is a daunting task and demands huge infrastructure and cost.

In this work, we have shown that hMSCs maintain their stemness over long passages when cultured on an optimally soft polyacrylamide (PAA) gel. The soft substrate also preserves cellular morphology. Staining for β-gal and BrdU respectively showed that in these cells onset of senescence is delayed and proliferative potential is maintained. Staining for other senescence-related changes such as loss of Lamin B and gain of Lamin A confirmed this observation. Not only the proliferative potential but the cells cultured on gel could differentiate into the adipo lineage, as shown by the expression of PPAR-gamma and accumulation of oil droplets, while cells cultured on tissue culture plastic (TCP) lose their adipogenic differentiation potential. Finally, we have shown that surface markers, used to characterize MSCs, remain unaltered in the cells cultured on soft substrate ensuring the maintenance of cellular identity.

## RESULTS AND DISCUSSION

### Loss of cell morphology and induction of senescence during long-term *in vitro* expansion

**Fig. 1.**
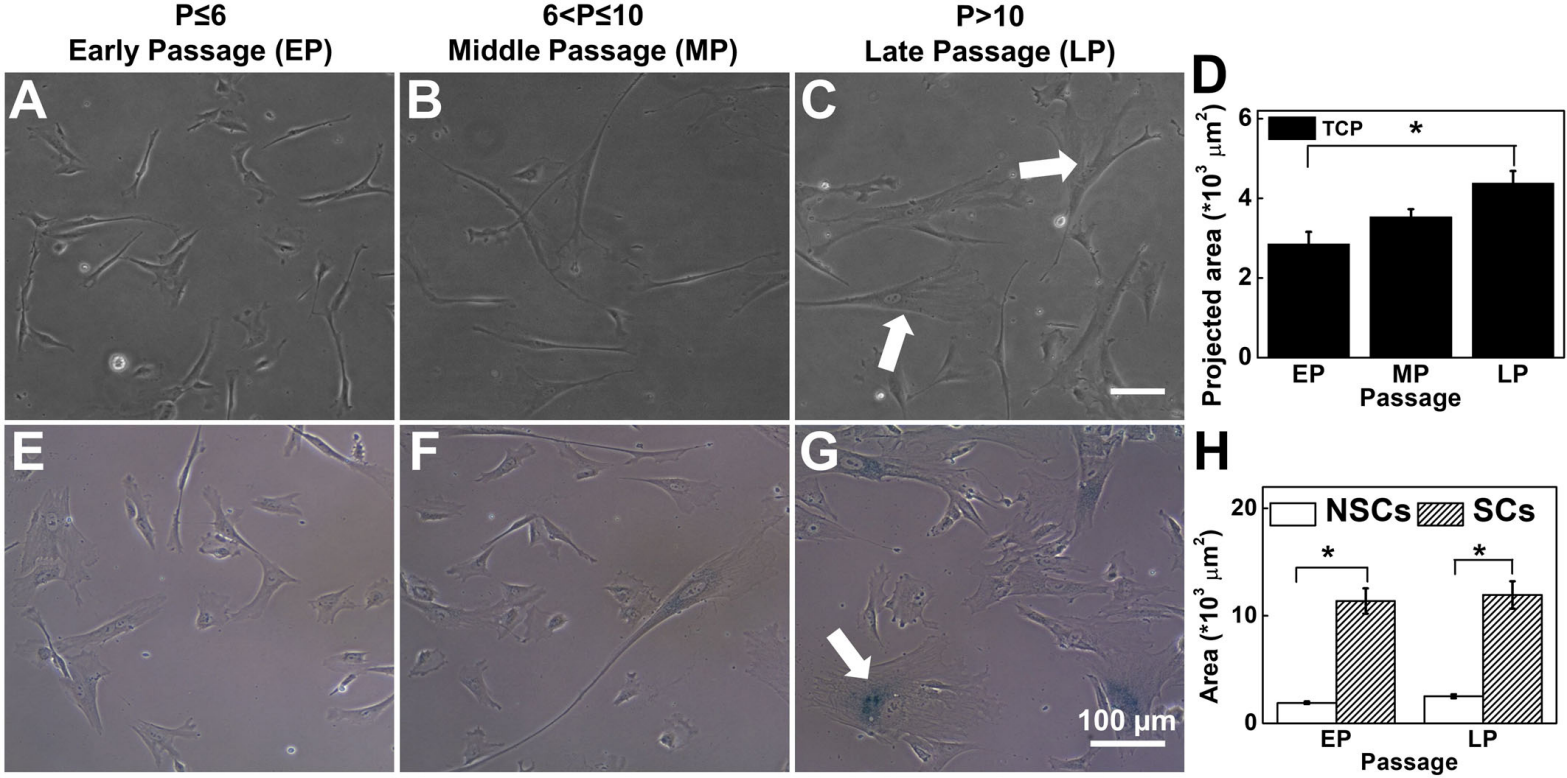
**hMSCs lose their morphology and enter into replicative senescence on long-term passaging on TCP.** Representative micrographs from (A) EP (passage number ≤6) (B) MP (passage number >6 and ≤10) and (C) LP (passage number >10) show that during *in vitro* expansion, MSCs lose their spindle morphology and become large and flat. White arrows show large cells with irregular shapes. (D) Over passage, average cell-spread area increases significantly from ∼3000–4500 µm^2^. (*N*=3, *n*=at least 150). (E–G) β-gal staining (a senescence marker) significantly increased in MP and LP cells compared to EP cells. White arrow indicates β-gal positive cells. (H) Spreading area of senescent cells and non-senescent cells: senescent cells have more spreading area than non-senescent cells, irrespective of passage (*N*=3, *n*>100). Results are expressed as mean±s.e.m., *P<0.05. Scale bars: 100 µm.

To study the effect of substrate stiffness on maintenance of stemness, we cultured umbilical cord-derived hMSCs (UC-hMSCs) on polyacrylamide gel and on TCP, both coated with collagen I, from passage 3 (P3) to passage 13 (P13) (Fig. 1). These cells were well characterized (SI appendix, Fig. S1) and applicable bio-safety and ethical guidelines were followed. For better understanding of the long-term effect of passaging on cellular behavior, we grouped our results as ‘early passage’ (EP), ‘mid passage’ (MP), and ‘late passage’ (LP), which were defined as passage number (*P*≤6), (*P*=7–10), and (*P*>10), respectively. This classification, though arbitrary, was done based on the prevalent practice that MSCs are generally used till maximum P6 for research and clinical applications (Binato et al., 2013; Bonab et al., 2006).

To measure spread area, cells were imaged after 24 h of cell seeding and average cell area was computed considering at least 150 randomly selected cells for each passage. We found that when cultured on TCP, UC-hMSCs with increasing passage lose their spindle morphology (Fig. 1A–C) and go into replicative senescence (Fig. 1E–G). The majority of the cells became flat and took irregular shapes with the passage as shown by the white arrows in Fig. 1C*.* The change in the projected area is quantified in Fig. 1D. Also, more debris and more granularity in the cytoplasm were observed for later passage cells (data not shown). To check the onset of senescence, we trypsinized the cells from their respective substrates and re-plated them on glass coverslips. After 24 h, we stained the cells with SA-β-gal, a well-established method to capture the senescent cells. We observed that while for EP cells only very few cells (<5%) were β-gal positive (Fig. 1E), it increases gradually to finally reach at about 20% for LP cells (Fig. 1G). Further, to check if the increase in the cell area and increased senescence are correlated, we estimated the average spread area of β-gal positive and β-gal negative cells for EP as well as for LP. We found that irrespective of passage number, senescent cells are always significantly larger compared to the non-senescent cells (Fig. 1H). Also, the average area of senescent and non-senescent cells remains almost unaltered over passage implying a possibility of close association of cell spreading with the onset of senescence. This observation led us to the hypothesis that maintaining cell size using soft substrates made of polyacrylamide gel may delay or stop senescence resulting in more efficient *in vitro* expansion of MSCs.

### Selection of substrate for further study based on cell morphology and proliferation

It is known from the literature that cells spread less and take a rounded morphology on soft substrates (Jalali et al., 2015; Tee et al., 2011). To find the suitable substrate that restricts cell spreading, we cultured LP cells on PAA gels of a stiffness range varying from 0.5–20 kPa and TCP (∼GPa) (SI Appendix, Fig. S2A–G). We found that on soft gels (*E*≤5 kPa), LP cells had a cell-spread area smaller than or equivalent to EP cells cultured on TCP (Fig. 1D and SI Appendix, Fig. S2G). This observation implied that the cell-spread area can be kept restricted over passages if cultured on soft gels (E≤5* *kPa). However the question remains, how soft can we go? To answer this question, we need to consider another parameter, i.e. the effect of substrate stiffness on cell proliferation. It is known that soft gel induces reversible cell cycle arrest or quiescence in hMSCs (Rumman et al., 2018; Winer et al., 2009). As a result, the very soft gel cannot be used for cell number expansion. To find the optimum range of stiffness, we cultured cells on substrates of different stiffness. After 48 h of culture on these gels, which is sufficient to induce quiescence (Rumman et al., 2018), we gave a 4 h pulse of BrdU that tags the replicating DNA. We found that while cells on 1 or 2 kPa gel showed critically less replicating DNA, cells on gels of 5 kPa and higher stiffness had more than 30% of dividing cells which is equivalent to that on TCP (SI Appendix, Fig. S2H–L).

Putting these two observations together, we selected 5 kPa gel for all our ongoing studies to compare the effect of substrate stiffness on long-term *in vitro* culture.

### Soft substrate maintains cellular morphology and self-renewal ability of UChMSCs

**Fig. 2.**
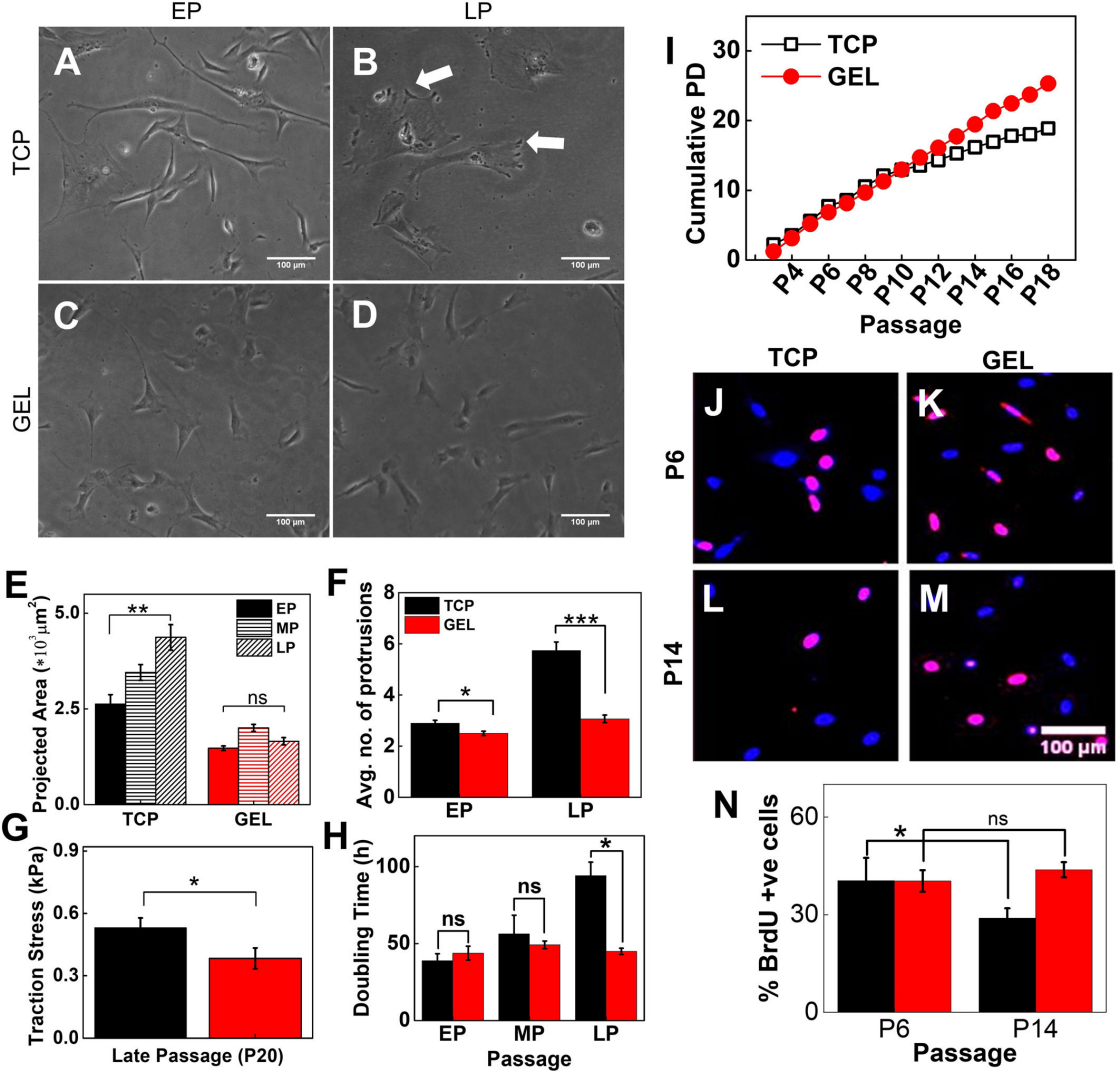
**Soft substrate maintains cellular morphology, rate of expansion and proliferation during serial passage.** Representative phase contrast images of (A) EP (passage number ≤6) and (B) LP (passage number >10) UC-hMSCs on collagen-coated TCP and (C) EP and (D) LP on soft gel. (E) Cell spread area increases with passage on TCP; however, spreading area of cells on gel showed no significant difference across passages. (*N*=3, *n*=150). (F) The number of protrusions, as shown by white arrows in B, are significantly increased in LP cells cultured on TCP (*N*=3, *n*=150). (G) LP UChMSCs on gel showed significantly lower traction than that on TCP. The experiment was performed using two biological samples (*N*=3, *n*=20). (H) DT of UC-hMSCs cultured on gel and TCP over the passage. While DT increases with passage when cultured on TCP, it remains unaffected when expanded on 5 kPa gel. The difference in DT for EP and MP are negligible while for LP the difference is significant (*N*=3, *n*=1). (I) CPD of UC-hMSCs cultured on gel and TCP over the passage. CPD increases linearly for UChMSCs on the gel but approaches a plateau for cells cultured on TCP (*N*=1, *n*=11). (J) Immunofluorescence images of nuclei co-stained with DAPI (blue) and BrdU (red) capturing a relative percentage of cycling cells. (K) Percentage BrdU positive cell for P14 (LP) from gels are significantly higher than the cells from TCP, though for EP (P6) cells there was no significant difference between gel and TCP. This data reconfirms that cells maintain their proliferative potential if cultured on the soft gel. Results are expressed as mean±s.e.m. *P<0.05, **P<0.001, ***P<0.0001. Scale bars: 100 µm.

When continuously cultured on 5 kPa gel for 45 days, it was observed that UChMSCs maintain their cellular morphology and proliferative potential better than the cells cultured on TCP. For P3 to P18, the cells were trypsinized in every 72 h and re-seeded on the respective substrates at 1000 cells/cm^2^ seeding density. Fig. 2A–D show that while cells lose their morphology both in terms of size and shape when cultured on TCP for multiple passages, they maintain the same morphology if cultured on the gel. The average area of cells on 5 kPa gel was maintained with the passage at about 1500 µm^2^, but the same was increased from 3000 µm^2^ to 4500 µm^2^ on TCP (Fig. 2E). On the gel we observed more spindle-shaped cells and fewer irregular protrusions, as can be seen in Fig. 2A–D and F. The same observations were made when the cells from EP. MP, and LP from both gel and TCP were trypsinized and seeded on to the plastic (Fig. S3). To have a blind test, we showed the cells under the microscope to multiple independent observers who were well versed with hMSCs morphology. They could not differentiate between EP and LP cells from the gel. However, the difference between EP and LP on TCP was obvious. Other than spread area and protrusions, cells expanded on the gel for LP also showed significantly lower traction (Fig. 2G) than the cells on TCP when tested on 5 kPa gel for traction force microscopy (TFM).

To check if along with maintenance of morphology, self-renewal efficiency is also maintained, we assessed the effect of a soft substrate on cellular expansion of UC-hMSCs in the long-term passage. Using microscopic images, as described in the Materials and Methods section, we counted cell number twice; first, after 4 h of seeding and second, just before harvesting. From the number of cells seeded and harvested we calculated population doubling (PD), doubling time (DT) and cumulative population doubling (CPD) as described in the Materials and Methods section. Fig. 2H compares DT values for EP, MP, and LP cells. We found that the DT for cells on TCP increased slightly from 40–50 h for EP to MP and then jumped significantly to more than 80 h for LP cells. However, the DT value remains almost constant for all three groups of cells (∼40 h) when cultured on 5 kPa PAA gels.

This observation is also reflected in the CPD (Fig. 2I). We observed that CPD on TCP increased almost linearly till P9 and then gradually slowed down. For gel, CPD continues to increase linearly till the end of the experiment. By P18, we observed a difference of 9 in CPD signifying that 2^9^ or 512 times more UChMSCs can be obtained from gel than from TCP. This difference signifies that while one single cell will give us 4 million cells after 18 passages if cultured on TCP, the same cell will give 2000 million or 2 billion cells if cultured on the soft gel.

Further, to confirm that this reduction in CPD on TCP compared to that on the gel is indeed due to the maintenance of proliferation and not a cell culture artifact, we performed BrdU assay to estimate the fraction of cells in *S* phase after harvesting them from their respective substrate and then reseeding on the glass. We found that there was no significant difference in the percentage of *S* phase cells between TCP and gels for EP population. However, for LP cells, the same was significantly higher when cells were harvested from the gels than their counterparts harvested from TCP (Fig. 2J–N). This data conclusively proves that 5 kPa PAA gel maintains self-renewal ability of UC-hMSCs over long-term culture leading to a significant increase in cell number. To rule out the possibility of higher cell adhesion on gel compared to TCP resulting into higher CPD, we checked for plating efficiency on both the substrates and found no significant difference (SI Appendix, Fig. S4).

All the results mentioned here were done with technical triplicates. To check the reproducibility of the data, we used two independent biological replicates and MSCs derived from bone marrow. We found similar observations in all these cases, as shown in the SI Appendix and Figs S5–S9.

### Soft substrate delays senescence

**Fig. 3.**
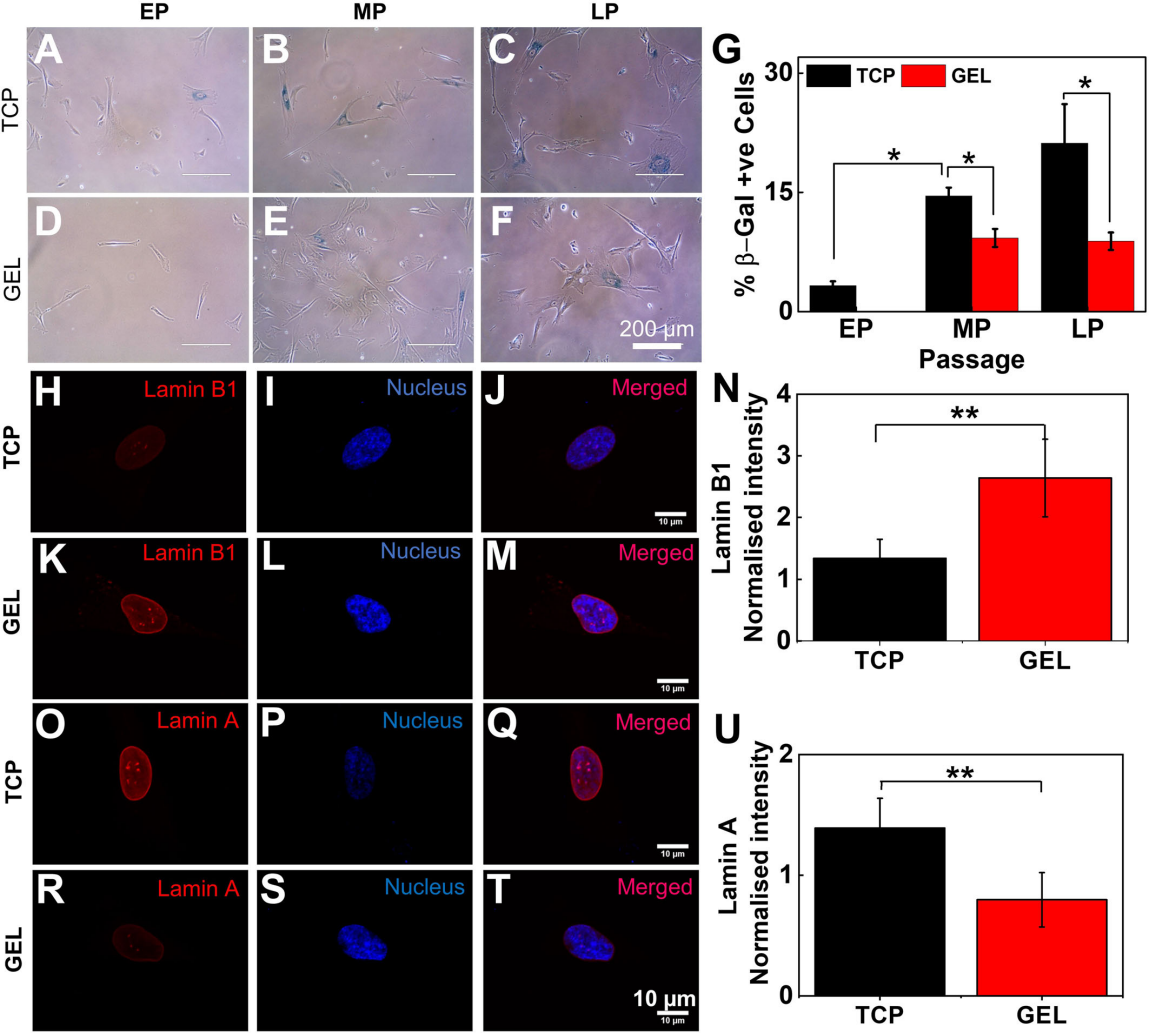
**Soft substrate delays senescence.** (A–G) Cells stained for β-gal (blue), a well-established senescence marker, show that while senescence gradually increases for TCP over the passage, on the gel after an initial increase it remains constant (EP: passage number ≤6, MP: passage number >6 and ≤10, LP: passage number >10). The same is quantified in G (*N*=3, *n*=400). Results are expressed as mean±s.e.m. *P<0.05. Scale bars: 200 µm. (H–N) Comparison of expression of total nuclear Lamin B1 (red) between LP UC-MSCs grown on TCP and gel. The decrease of Lamin B1 expression on TCP (H–J) compared to UC-hMSCs on the gel (K–M). Quantification of intensity shows that Lamin B1 is maintained in late passage UC-hMSCs on the gel (N) (*N*=2, *n*=22). Further, comparison of Lamin A expression for late passage UC-hMSCs on plastic (TCP) (O–Q) and on the gel (R–T) reveals the fact that Lamin A expressed more on TCP. The same is quantified in figure (U) (*N*=2, *n*=20). Results are expressed as mean±s.d. **P<0.001. Scale bars: (H–T) 10 µm.

To verify if this maintained rate of expansion on soft gels is a result of reduced senescence as we proposed earlier, we stained the cells with SA-β-gal, a standard technique to estimate senescence. We found that on TCP, the fraction of senescent cells increases with the passage, reaching >20% of the population for late passages (Fig. 3A–C,G). However, when cultured on the gel, although there was an increase in senescent population from EP to MP, the percentage of this population did not increase further and remained <10%, which is significantly less compared to the same for cells cultured on TCP (Fig. 3D–G). To reconfirm, we also checked for expression of vimentin, which is known to overexpress in senescence fibroblast (Frescas et al., 2017; Nishio et al., 2001). Immunofluorescence analysis revealed a fourfold increase in vimentin expression in cells cultured on TCP compared to the same on gel (SI Appendix, Fig. S10).

We also checked for Lamin A and Lamin B1, two nuclear envelope-associated proteins, expression of which is known to vary differentially in senescence. Loss of Lamin B1 and accumulation of Lamin A are known to increase in senescent cells and are used as novel biomarkers for senescence (Bellotti et al., 2016; Freund et al., 2012; Wang et al., 2017). To investigate the expression of Lamin A and Lamin B, we trypsinized cells from gel and TCP and plated them on glass coverslips. After 24 h of cell seeding, we stained the cells with respective antibody and quantified Lamin A and B1 expression for the whole nucleus. We indeed found that expression of Lamin B goes down (Fig. 3H–N) and Lamin A goes up (Fig. 3O–U) for cells from TCP compared to the cells harvested from gels. Both of these observations, along with SA-β-gal staining and vimentin expression, show that culturing UC-hMSCs on soft gel delays senescence.

### Long-term culture on gel did not alter surface marker expression but helps the stem cells to maintain differentiation potential

**Fig. 4.**
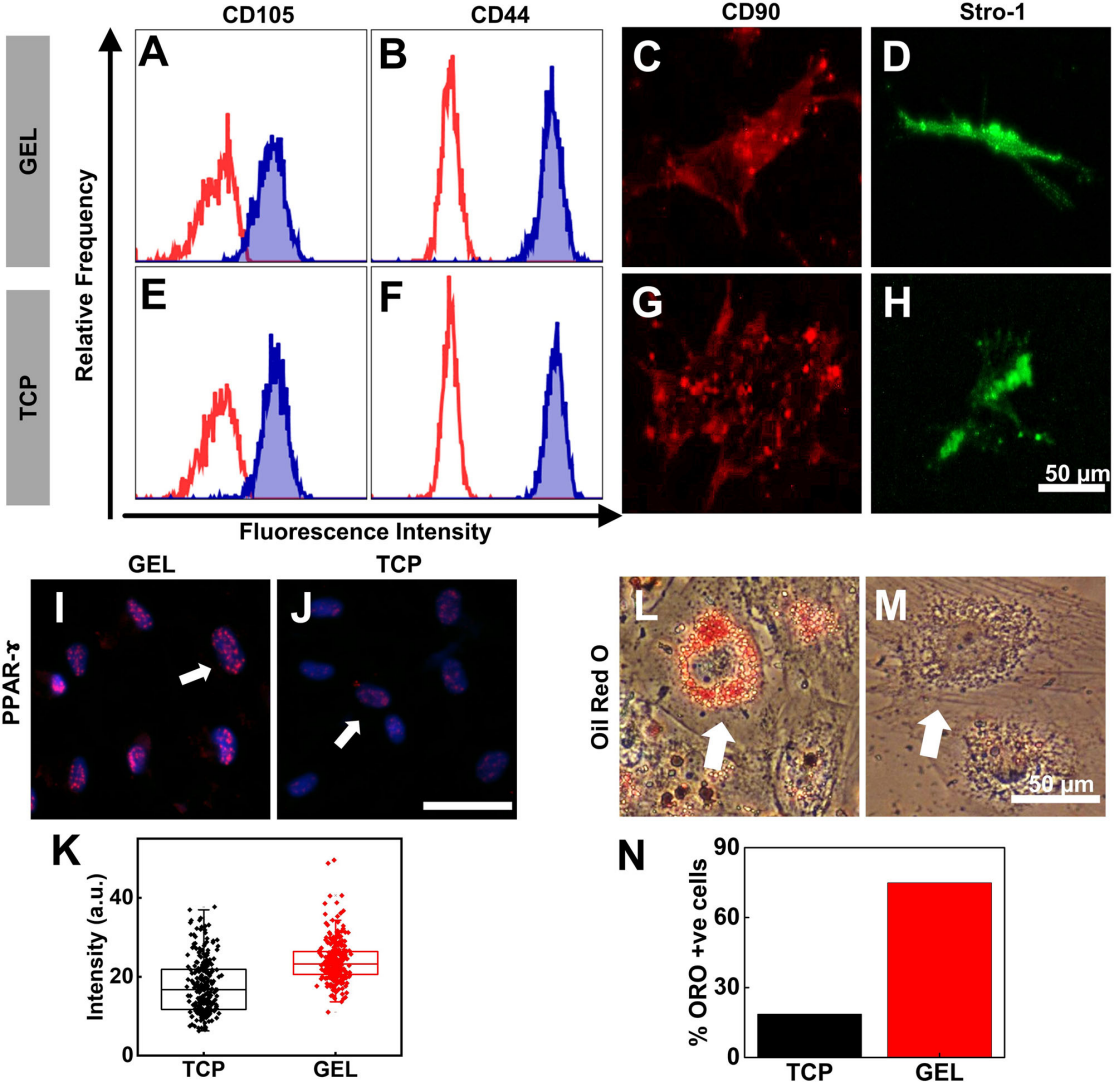
**Long-term culture on gel did not alter surface marker expression but helps stem cells maintain differentiation potential.** Flow cytometry analysis of the expression of surface pluripotency markers of UC-hMSCs at the LP (P22) from the gel (A–D) and TCP (E–H) was determined. The expression of surface markers, CD105, CD44, CD90, and Stro-1 was not altered by the long-term culture of UChMSCs on the gel. The red curve in A, B, E and F is auto-fluorescence of the cells and the blue filled histogram is the fluorescence signal of the stained marker. (I–N) Adipogenic differentiation: LP hMSCs (P14) from gel and TCP were cultured in adipogenic induction media. (I–K) The early adipogenic marker PPAR-γ expression was checked after 7 days of adipo-induction using immunostaining of UC-hMSCs from gel (I) and TCP (J) (magenta puncta in the blue nucleus, shown by white arrow). The UC-hMSCs serially passaged on gel showed higher expression of PPAR-γ compared to the cells cultured on TCP, as quantified in K (*N*=2, *n*=273, ***P<0.001). (L–N) After 14 days of adipogenic induction, the lipid droplets accumulation was significantly higher in cells from the gel (L) than from TCP (M). Lipid droplets were identified by staining with Oil Red O. The percentage of Oil Red O-positive cells was higher on the gel substrate compared to TCP, as quantified in N (*N*=2, *n*=100). White arrow shows cells with and without oil droplet accumulation. Scale bars: 50 µm.

Finally, to confirm the identity of our cells after long-term culture, we investigated the effect of substrate on the expression of surface pluripotency markers. Surface marker analysis of late passage (P22) UChMSCs using flow cytometry and immune-cytometry demonstrated that cells cultured either on gel or on TCP express characteristic positive surface markers, CD105 (Fig. 4A,E), CD44 (Fig. 4B,F), CD90 (Fig. 4C,G) and Stro-1 (Fig. 4D,H). This is in accordance with the previous studies which showed that the MSC surface marker expression remains same with increasing passage (Kundrotas et al., 2016). However, the difference appears in their differentiation potential. While LP cells on TCP lose their adipogenic potential, LP cells on gel did not (Fig. 4I–N). We harvested the cells from their respective substrates and cultured them on TCP in adipogenic induction media for 7 days. Then the cells were fixed and stained for the early adipo transcription factor, PPAR-γ. It is evident from the images that LP cells from gel expressed a much higher level of PPAR-γ than the LP cells from TCP in similar condition (Fig. 4I–K). The same difference was observed after 14 days of adipo induction in terms of accumulation of oil droplets that were detected with Oil Red O dye (Fig. 4L,M). Approximately 75% of LP cells on gel cells differentiated into adipocytes as determined by ORO in comparison to only 18% of LP cells on TCP cells staining (Fig. 4N).

In this work, we have demonstrated that a soft PAA gel substrate of 5 kPa elastic modulus can maintain the stemness of UChMSCs *in vitro* by delaying the senescence process. Like any other primary cells, hMSCs undergo only a certain number of divisions before entering into replicative senescence. Replicative senescence is typically marked by large and flat morphology, and significantly slowed down proliferation rate (Banfi et al., 2000; Wagner et al., 2008). Multi-lineage differentiation potential also goes down with population expansion (Bonab et al., 2006; Wagner et al., 2010b). This decreased proliferation and loss of multi-lineage potential poses a serious challenge in using *in vitro* expanded hMSCs for clinical use. In this work we have shown that culturing UChMSCs on soft substrate maintains self-renewal ability, multi-lineage potential and surface markers much beyond the same when cultured on TCP.

As previous studies have shown that the span of culture before the onset of a senescence program can vary significantly depending on cellular origin, we first cultured our UChMSCs on TCP up to 13 passages and confirmed the onset of senescence with enlarged and flat morphology, and β-gal staining (Fig. 1) (Bonab et al., 2006; Vidal et al., 2012). We also showed that average area of non-senescent cells remains same over passages. Recently, Neurohr et al. showed that when cells grow too large, DNA required for proper cellular functions becomes limiting, leading to senescence (Neurohr et al., 2019). This observation opens up a possibility that restricting cell spreading may help in delaying senescence. Such hypothesis is supported by work from Killian's group showing that MSCs cultured on micro-patterned substrates to restrict cell spread area helps in maintaining the expressions of stemness markers STRO-1 and Endoglin (Zhang and Kilian, 2013; Lee et al., 2015). Although these results point towards the fact that stemness can be maintained by culturing cells on micro-patterned island thus restricting spreading, such approach is not very useful for MSC expansion as cells on micro-island stop proliferation and do not increase in number.

Earlier studies also showed that stemness in hMSCs could be maintained better by reducing actomyosin contractility or cellular traction using pharmacological inhibitor of ROCK and myosin (Zhang and Kilian, 2013). In a different study, it was shown that while mESCs (mouse embryonic stem cells) grown on TCP need LIF to stop spontaneous differentiation, a very soft substrate (600 Pa) can maintain them in their undifferentiated state without LIF (Chowdhury et al., 2010). All these results indicated that cell area/contractility plays an important role in the loss of stemness and both can be kept low if cultured on a soft substrate (McBeath et al., 2004; Tee et al., 2011). However, none of these works checked the effect of substrate stiffness in long-term culture. We demonstrated here that a long-term culture on soft substrate may inherently reduce the cellular traction (Fig. 2G) and thus can maintain stemness. To the best of our knowledge, this is the first work to demonstrate the effect of substrate stiffness on cellular traction and maintenance of stemness in long-term culture (for 20 passages i.e. ∼60 days) for any cell type.

Consistent with the previous report, we also found that though UC-hMSCs lose their self-renewal ability when cultured on TCP, they maintain the molecular signatures related to stemness (Fig. 4A–H) (McGrail et al., 2013). The cells, irrespective of cultured on gel or TCP were positive for CD105, CD44, CD90, and Stro-1. However, cells cultured on TCP lost their adipogenic differentiation ability whereas the same was maintained for the cells cultured on the gel (Fig. 4I–N). Loss of adipogenic potential over long term passages and dominance of osteogenic differentiation has been reported by many earlier researchers (Neuhuber et al., 2008; Wall et al., 2007). It was shown that flat cells that appear spontaneously over long-term culture, lose their adipogenic potential (McGrail et al., 2013). This observation is not unexpected if we look from the cell mechanics angle. It was established by Engler et al*.* in their seminal paper in 2006 that stiff substrate (34 kPa) induces osteogenic lineage in hMSCs (Engler et al., 2006) even in absence of chemical inducer. Similarly, it was also shown that cells that were made to spread more or to take the shape that induces high contractility, also were prone to osteogenic lineage commitment (Kilian et al., 2010; McBeath et al., 2004). So, it is expected that if for multiple passages, cells are continuously exposed to a substrate as rigid as TCP which increases cellular spreading and contractility, adipogenic potential would get diminished. However, a soft culture substrate, in contrast, should maintain the multi-lineage potential, as demonstrated by our result (Fig. 4).

Other than UChMSCs, we also used bone marrow-derived hMSCs and found a similar result proving that this effect might not be source specific. We have also found that the substrate stiffness for optimal growth of skin-derived keratinocytes is not the same as for MSCs (data not shown). How cell type and optimum substrate stiffness are inter-linked is open for future investigation.

One of the interesting observations in this work is that soft substrate delays senescence. It is known that acquiring replicative senescence over *in vitro* expansion may not be an obvious purposeful program but a result of the external environmental condition. For example, it has been shown that increased oxygen concentration may induce senescence faster. On the contrary, the hypoxic condition is known to maintain stemness for hMSCs (Basciano et al., 2011). However, the effect of substrate stiffness on senescence has not been studied before. We have demonstrated using four known markers of senescence – namely expression of β-gal, loss of Lamin B1, the gain of Lamin A and vimentin (Bellotti et al., 2016; Bonab et al., 2006; Freund et al., 2012) – that an optimally soft substrate may delay the onset of senescence significantly.

In summary, our data show that instead of using TCP, culturing cells on the soft substrate will help to solve the problem of limited availability of MSCs by increasing the number of available cells after extended expansion. This work offers a possibility to design a cell-specific culture substrate in the future. This work also demonstrates for the first time that replicative senescence in hMSCs can be delayed using substrates of physiological stiffness.

## MATERIALS AND METHODS

### Substrate preparation

Gels of polyacrylamide (PAA) of various stiffness were prepared by mixing 40% polyacrylamide and 2% bis-acrylamide solution, as described previously (Pelham and Wang, 1997). Substrate preparation protocols and modulus values were adopted from previously published work (Tse and Engler, 2010). Briefly, the gel solution for desired stiffness was mixed with APS (ammonium persulfate) 1:100 and TEMED (1:1000) and placed between a hydrophobic glass (octadecyltrichlorosilane treated; Sigma-Aldrich, 104817) and the transparency sheet 3-APTMS (Alfa Aesar, A17714) treated. Once polymerized, the hydrophobic plate was carefully removed. The gel was conjugated with sulfo-SANPAH and incubated with rat tail type I collagen (25 µg/ml) (Invitrogen, A1048301) at 4°C for overnight, as described (Venugopal et al., 2018). The tissue culture plates (TCP) (control) were also coated with type 1 collagen (25 µg/ml). The thickness of the gel was controlled by using the defined volume of the gel solution throughout the experiments.

### Cell culture

Bone marrow-derived human MSCs were purchased from Lonza (Cat #PT-2501, Lot #482966) (authenticated and tested for contamination by the supplier), and fresh umbilical cord-derived MSCs were obtained from healthy individuals after due ethical clearance and bio-safety approval. For serial passage experiments, P4 cells were seeded on large area gels and on TCP (both collagen-I coated as mentioned above) with same seeding density (1000 cells/cm^2^) in MSCs qualified medium α-(MEM) (Invitrogen, A1049001). Low-glucose DMEM (HiMedia, AL006) supplemented with 16% MSC certified fetal bovine serum (FBS) (Invitrogen, 12662029), 1% Glutamax (Invitrogen, 35050061), and 1% pen-strep (Invitrogen, 15140122) in humidified incubator with 37°C and 5% CO_2_. After 72 h of culture, cells were trypsinized from PAA gels and TCP using TrypLE™ Enzyme Express (Invitrogen, 12604013) and were reseeded on fresh substrates respectively and cultured for next passage, this process was repeated until the TCP growth halted.

### Cell count using image analysis

Images of the gels and TCP were acquired using a Magnus microscope at 10× magnification after 4 and 72 h of seeding to determine accurate cell number for calculating PD as described (Cristofalo et al., 1998). For PD counting, 20 random images per sample were captured (covering ∼3% of the total area of the gel), the average number of cells per frame was obtained and then divided by the total area of the frame to obtain seeding density (cells/cm^2^). The seeding density was then multiplied by the total area of the substrate (gel 20 cm^2^; TCP 25 cm^2^) to get the total number of cells seeded (4 h) and harvested (72 h) from a particular experimental condition (PAA gels and TCP) for respective passage. This was done in every passage, which was then used to calculate the CPD for each experimental condition as explained in the Eqns. 1–3 (Cristofalo et al., 1998):(1)
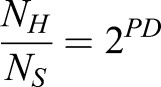
(2)
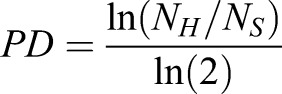
where *N_H_* is the number of harvested cells, *N_S_* is the number of cells seeded,(3)
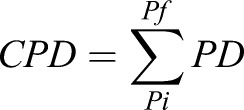
where *Pi* is the initial passage number and *Pf* is the final passage number.

### Quantification of cell morphology

Cell images were captured at different passages at 48 h post seeding using EVOS-FL auto inverted microscope (Life Technologies) at 10× magnification. Cell spreading area was determined using ImageJ (National Institutes of Health) software by manually tracing around the perimeter of an individual cell. For each sample minimum, 150 random cells were analyzed. The number of protrusions of cells was quantified from phase contrast images manually using ImageJ.

### Plating efficiency

To compare the cell adhesion efficiency, P9 bone marrow MSCs were seeded on the 5 kPa gel and collagen-coated coverslip. After 15 min of seeding, 15 random images were captured and analyzed to get the number of cells seeded. The plate was kept in the incubator for 1 h and 30 min, then media was aspirated and fresh media was added in each well. Again, 15 random images were captured and analyzed to determine the number of cells attached. From the number of cells seeded and the number of cells attached, we calculated the plating efficiency as:(4)
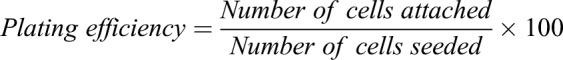
100% plating efficiency shows all cells attached.

### BrdU assay

To check the percentage of S-phase cells in the cell cycle, cells from EP, MP and LP were trypsinized from gels and TCP and were seeded on collagen-coated glass coverslips as described above. After 48 h of seeding, BrdU reagent (Invitrogen, 000103) was added in 1:100 (v/v) ratio in media and incubated for 4 h at 37°C in a humidified incubator with 5% CO_2_. Thereafter cells were fixed (4% paraformaldehyde), permeabilized (0.5% Triton-X), denatured (2 M HCl), blocked (1.5% bovine serum albumin), and incubated with anti-BrdU antibody (Invitrogen, B35128, 1:100) and counterstained with AlexaFluor 568 (Invitrogen, A11061, 1:400). Immunofluorescence images were captured using EVOS-FL auto and BrdU positive and negative cells were counted manually using ImageJ.

### Senescence assays

Senescence-associated β-galactosidase (SA-β-gal) was used to detect MSCs senescence using SA-β-gal staining kit (Abcam, AB65351) according to the manufacturer’s instructions. Briefly, cells from EP, MP, and LP were seeded in a six-well plate and incubated in growth media for 48 h. Afterwards, cells were fixed, stained with β-gal solution and incubated at 37°C without CO_2_. Ten–15 random images were captured for each condition for analysis β-gal positive cells were counted manually.

### Differentiation assays

EP and LP cells from gel and TCP were seeded in a 12-well culture plate in growth medium for 72 h and then incubated with differentiation media for adipogenic (Invitrogen, A1006501) and osteogenic (Invitrogen, A1006601) differentiation as per the manufacturer's instructions. MSCs cultured in growth media were used as a negative control. Post 14 days and 21 days incubation for adipo and osteo differentiation, respectively, adipocytes were assessed with Oil Red O (Sigma-Aldrich, O0625) solution and osteoblasts were assessed with Alizarin Red solution (Sigma-Aldrich, 3422613022311). Images were captured for qualitative and quantitative analysis using EVOS FL Auto.

### Immunofluorescence staining

For nuclear Lamin A (Abcam, ab8980, 1:400), Lamin B1 (Abcam, ab16048, 1:400), and early adipogenic differentiation marker staining, EP and LP cells from gel and TCP were cultured on collagen-I-coated glass coverslips for 24 h. Cells were then fixed with 4% paraformaldehyde in PBS for 15 min at room temperature (RT) and blocked (3% bovine serum albumin in PBS) for 30 min and washed with cytoskeletal buffer, as described previously (Venugopal et al., 2018). Cells were incubated with respective primary antibodies for 4 h at 4°C, and then incubated with corresponding secondary antibodies for 1 h at RT. Primary and secondary antibodies were used in the following combinations: anti-PPAR-γ (Abcam, ab59256, 1:300) counterstained with AlexaFluor 488 (Invitrogen, A11034, 1:500), anti-Lamin A (Abcam, ab8980, 1:400) counterstained with AlexaFluor 568 (Abcam, ab175473, 1:400), anti-Lamin B1 (Abcam, ab16048, 1:400) counterstained with AlexaFluor 568 (Abcam, ab175470, 1:400), anti-Vimentin (Sigma-Aldrich, V5255, 1:300) counterstained with AlexaFluor 488 (Invitrogen, A11059, 1:500). Cell nuclei were stained with Hoechst 33342 (Invitrogen, H3570) (1:10,000) in PBS for 5 min at RT and mounted. Images were captured for qualitative and quantitative analysis using EVOS fluorescence microscope (Invitrogen).

### Traction force microscopy (TFM)

Gels of 5 kPa were fabricated with embedded fluorescent beads to conduct TFM. Briefly, to make a single layer of the fluorescent bead (Fluka, 1 µm rhodamine), beads (1:50) were added to the pre-polymer solution (25 µl) and solidified over the normal gel of 5 kPa. The gel was then functionalized as described above. Cells were seeded on the gels, after 24 h of cell seeding images of stressed (before lysing) and unstressed gel (after lysing with 1% Triton-X) were captured by the EVOS FL Auto (Invitrogen). An average of 20 cells were analyzed per condition. A MATLAB algorithm was used to determine the cell-generated displacement field and traction forces as previously described (Butler et al., 2002). The TFM data was analyzed using MATLAB R2018a (IIT Bombay License).

### Statistical analysis

Data is presented as means±standard error of the mean (s.e.m.). For statistical analysis, we used unpaired Student’s *t*-test and values of *P*<0.05 were considered statistically significant, if not otherwise stated. Data was plotted using Origin software (IIT Bombay License).

## Supplementary Material

Supplementary information
