# Supplementary material for: Soft substrate maintains proliferative and adipogenic differentiation potential of human mesenchymal stem cells on long-term expansion by delaying senescence
Source: Biol Open. 2019 Apr 25;8(4):bio039453. doi: 10.1242/bio.039453 (PMC6503999; doi:10.1242/bio.039453)
Supplement: Supplementary information [file biolopen-8-039453-s1.pdf]

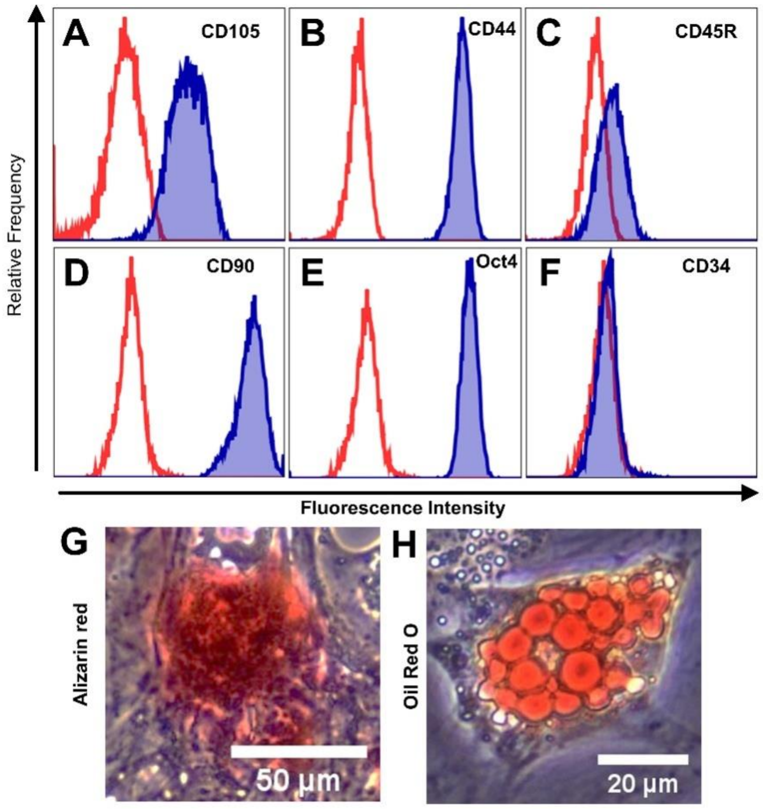

Figure S1: Characterization of hMSCs: Flow cytometry analysis for the expression of positive and negative markers of UC-hMSCs at early passage (P4) was determined (A-F). The expression of surface markers, CD105 (A), CD44 (B), CD90 (D), and pluripotency marker Oct4 (E) was positive whereas the cells did not express CD45R (C) and CD34 (F) which are negative markers for hMSCs. Red line in flow cytometry data is auto-fluorescence of cells and the blue filled histogram is the fluorescence signal from the marker. hMSCs were cultured in osteogenic and adipogenic induction media for 21 days and 14 days, respectively. Alizarin red staining was performed to identify the calcified nodules in the osteo-induced hMSC (G). The adipo-induced hMSC accumulated lipid droplets stained with Oil red O (H).

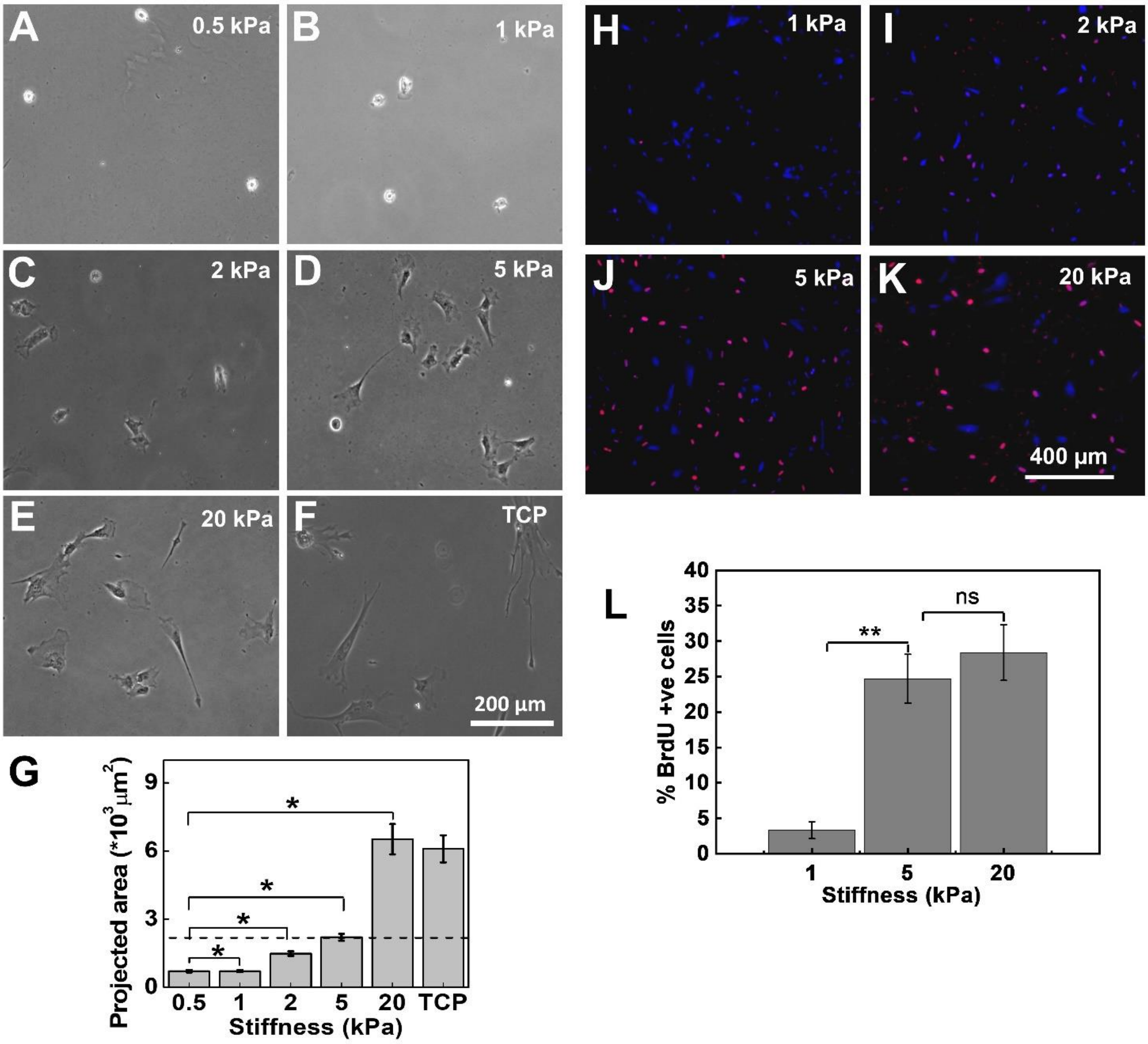

Figure S2: Soft Substrates restrict cell spreading and hinders proliferation: Phase contrast images of LP UChMSCs after 24 hours of seeding on substrates of various stiffness, (A) 0.5 kPa, (B) 1 kPa, (C) 2 kPa, (D) 5 kPa, (E) 20 kPa, and (F) plastic (TCP) show that cell spread area increases with substrate stiffness. (G) Quantification of average spreading of LP cells on various substrates shows that LP cells on gels with stiffness ≤ 5 kPa are even smaller than the EP cells on TCP. Average area of EP cells on TCP is shown by the dash line. (H-K) Representative images of BrdU stained nuclei on PAA gels of various stiffness (1 kPa, 2 kPa, 5 kPa, and 20 kPa) (Pink: BrdU positive nuclei, Blue: BrdU negative nuclei). (L) The quantification of BrdU show that very soft gel prohibits cell proliferation, hMSCs are more proliferative on gel of 5 kPa and above. Results are expressed in mean± s.e.m. Two tailed t-test *p<0.05, **p<0.002. (N=3, n = 100 cells for area analysis (G) and n > 250 nuclei for BrdU analysis (L)).

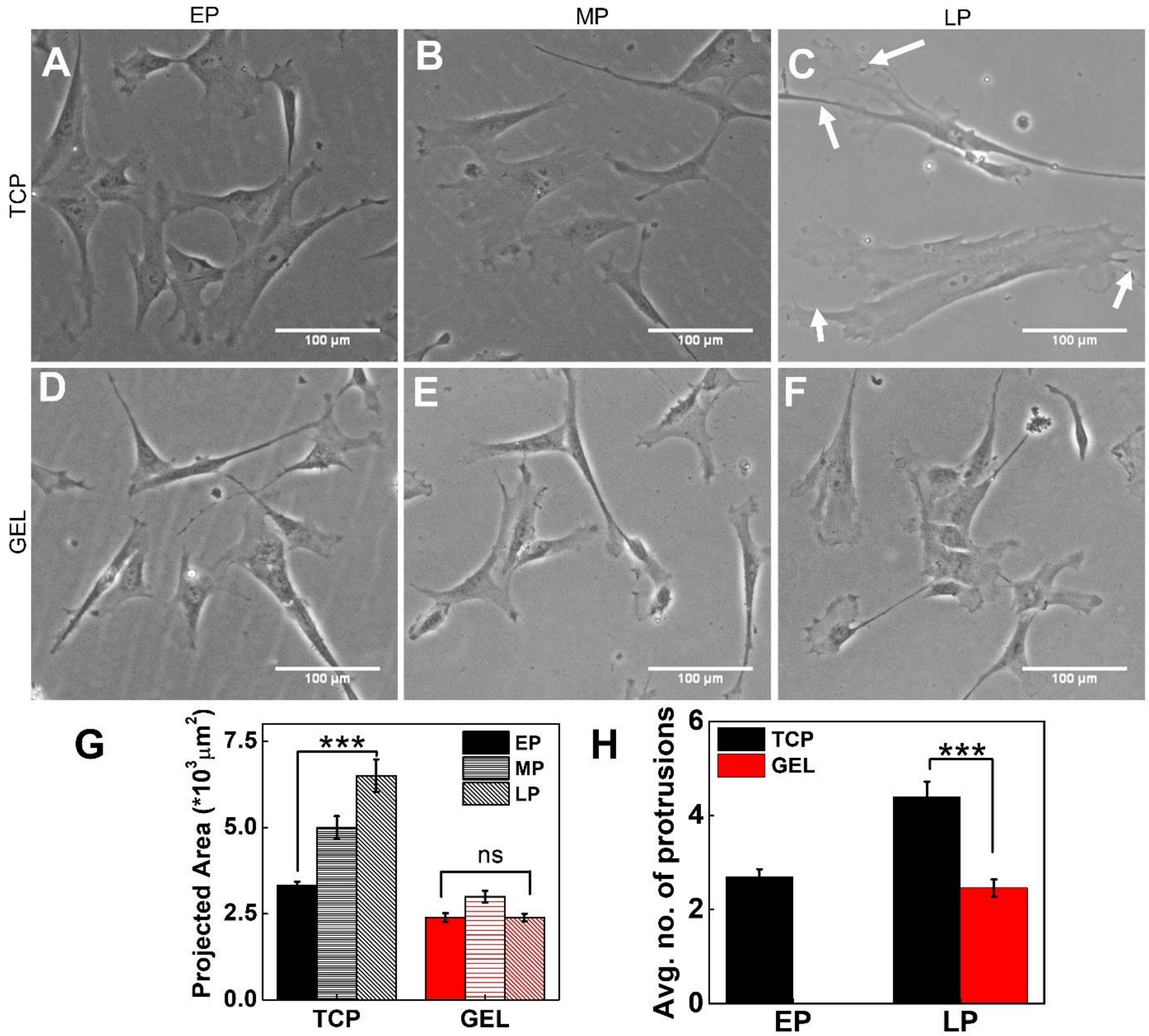

Figure S3: Soft gel preserved cellular morphology. Representative phase contrast images of (A and D) early, (B and E) middle and (C and F) late passage UC-hMSCs from (A-C) TCP and from (D-F) soft gel. The cells were trypsinized from their respective substrates and then reseeded on TCP. Images were taken after 48 h of reseeding on TCP. Scale bar=100 µm. (G) Quantification of cell area of EP, MP, and LP cells. Over passage, average cell spread area increases significantly from ~3000 μm^2 to ~6000 μm^2. Data shown is from three independent technical replicates as mean±s.e.m. (n>150). (H) Increased number of protrusions signifies loss of morphology. White arrows show the protrusion in late passage cells. Data shown is from three independent technical replicates as mean±s.e.m. Two tailed t-test ***p<0.0001. (n>30)

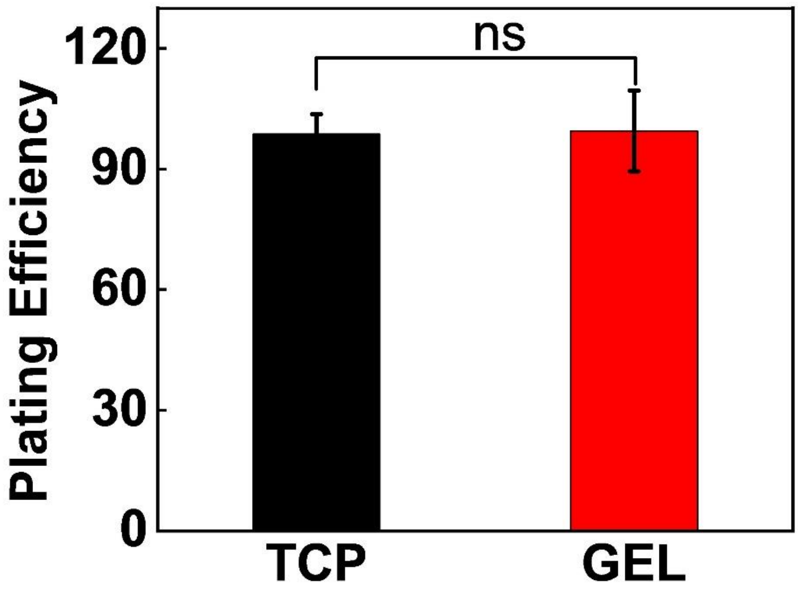

Figure S4: Comparison of Cell plating efficiency between 5 kPa gel and TCP shows that Gel and TCP have similar plating efficiency. Results are plotted as mean±s.e.m. N=3, Two tailed t-test with three technical replicates.

Biology Open • Supplementary information

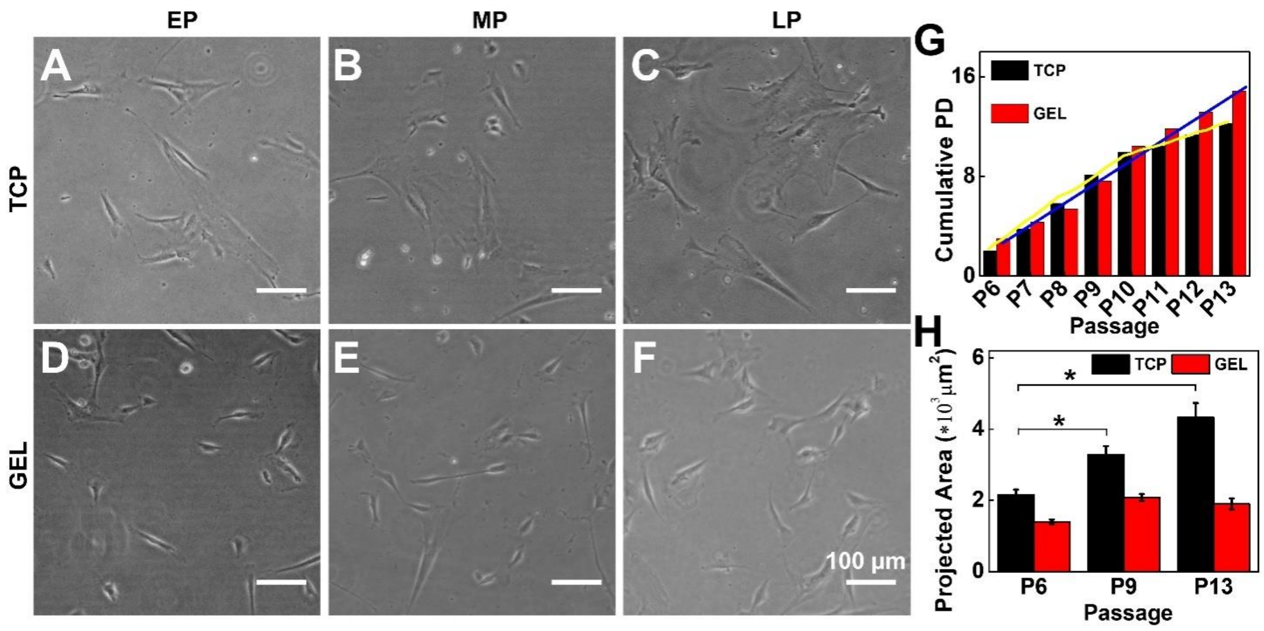

Figure S5: Biological repeat using UChMSCs from donor #02. Representative phase contrast images of (A) early, (B) middle, and (C) late passage UChMSCs of donor #02 on TCP and (D) early, (E) middle and (F) late passage cells on gel. (G) Cumulative PD (CPD) of UChMSCs cultured on gel and TCP. CPD increases linearly for UChMSCs on the gel but approaches the plateau for cells cultured on TCP. (H) Projected cell area increases from 2200 µm^2 for EP cells to 4400 µm^2 for LP cells on TCP whereas spreading remained 2000-2300 µm^2 on gel. Results are plotted as mean±s.e.m. Two tailed t-test *$p<0.002$ (N=3 and n=150).

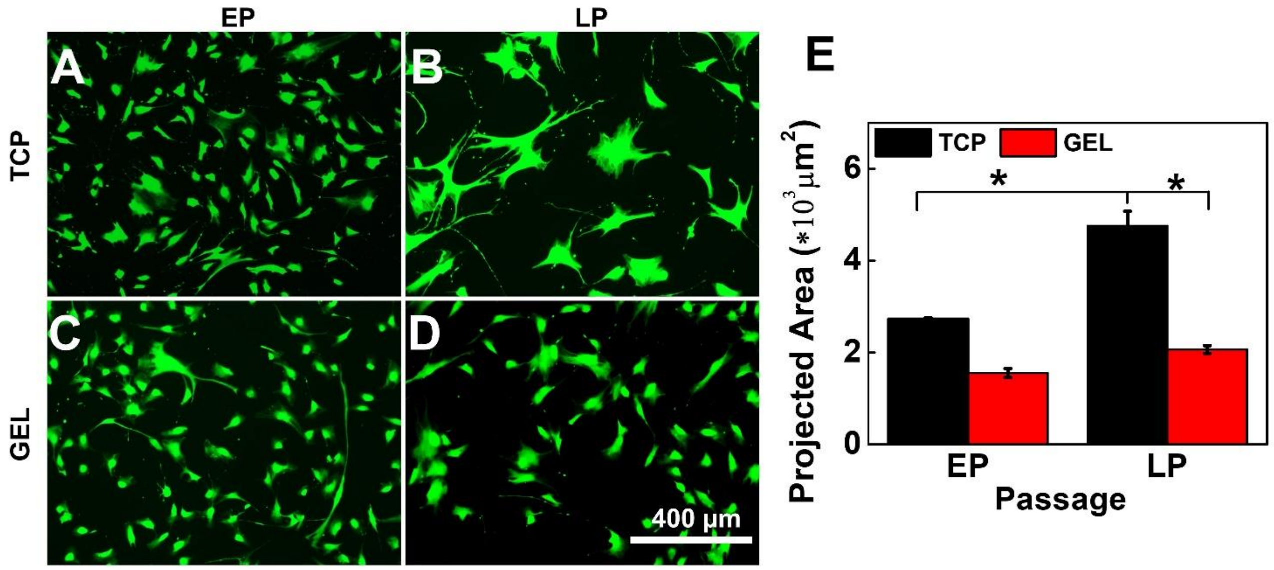

Figure S6: Morphological changes of bone marrow derived hMSCs. CalceinAM stained images of (A) EPand (B) LP cells after 24 h of seeding on TCP and (C) EP and (D) LP cells on gel. (E) Quantification of cell area data shows that soft gel restricts spreading while cell spread on TCP from 2500 to 5000 µm^2 even when cells were trypsinized from gel and seeded on TCP. Results are expressed as mean±s.e.m. Two tailed t-test *p value <0.05 (N=3 n=120).

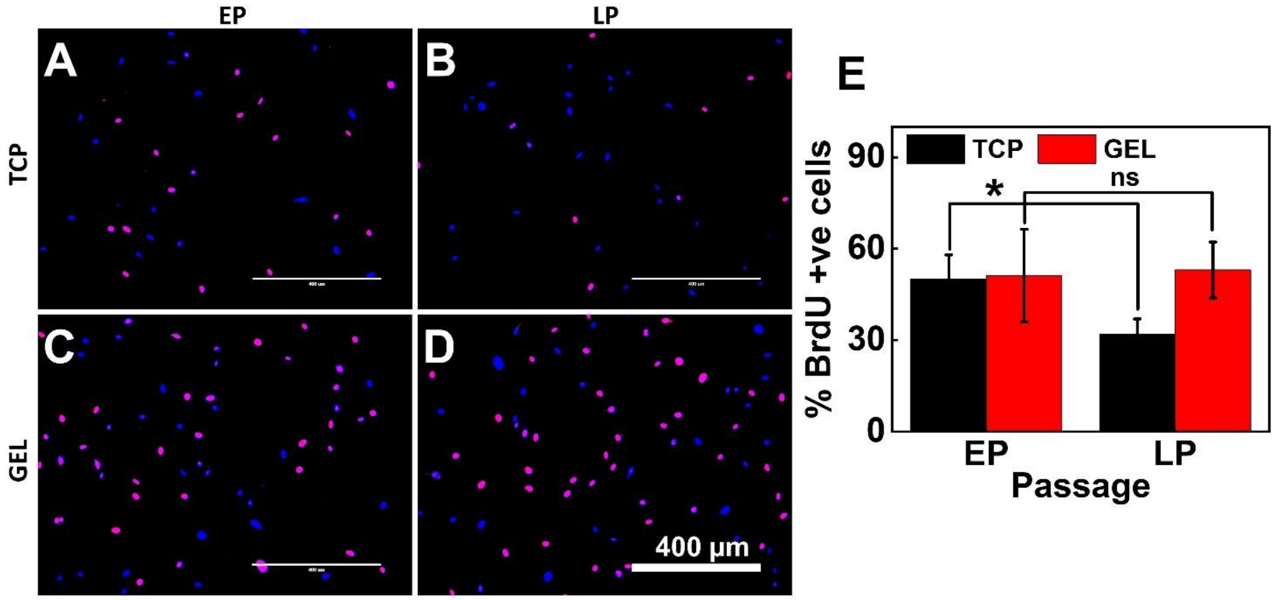

Figure S7: DNA replication of bone marrow derived hMSCs. BrdU incorporation in EP and LP cells on TCP (A, B) and gel (C, D). Blue and pink dots represent BrdU negative and positive nuclei, respectively. (E) Cell proliferation (% BrdU +ve cells) decreased with increasing passage on TCP but was maintained on gel. Results are expressed as mean±s.e.m. Two tailed t-test* $p<0.05$ (N=3, n=300).

Biology Open • Supplementary information

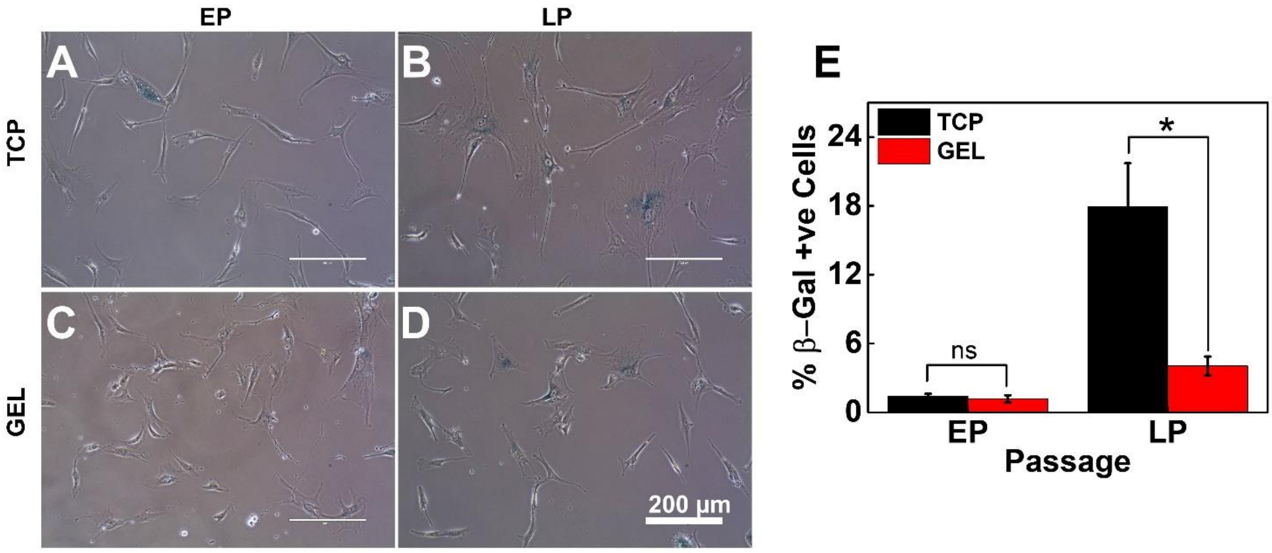

Figure S8: Replicative senescence of bone marrow derived hMSCs. Representative coloured images for (A) EP, (B) LP on TCP and (C) EP and (D) LP on gel stained for β-Gal (used to identify senescent cells). Blue coloured cells show β-Gal +ve senescence cells. (E) Quantification of β-Gal+ve cells shows a significant increase in number for LP/TCP compared to LP/Gel. Results are expressed as mean±s.e.m. Two tailed t-test *$p<0.05$ (N=3, n ≥ 150).

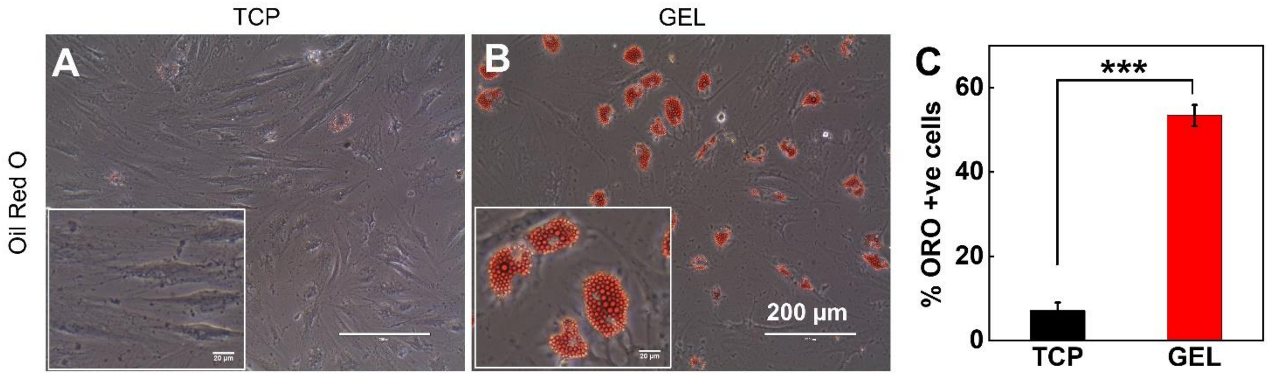

Figure S9: Differentiation potential of late passage bone marrow derived hMSCs. Oil red O stained images of cells from TCP (A) and gel (B) for accumulation of lipid droplets in late passage cells. (C) Quantification of Oil red O (ORO) was done by counting ORO positive cells manually. Results are expressed as mean±s.e.m. Two tailed t-test**$p<0.05$. ($N=5$, $n \geq 100$)

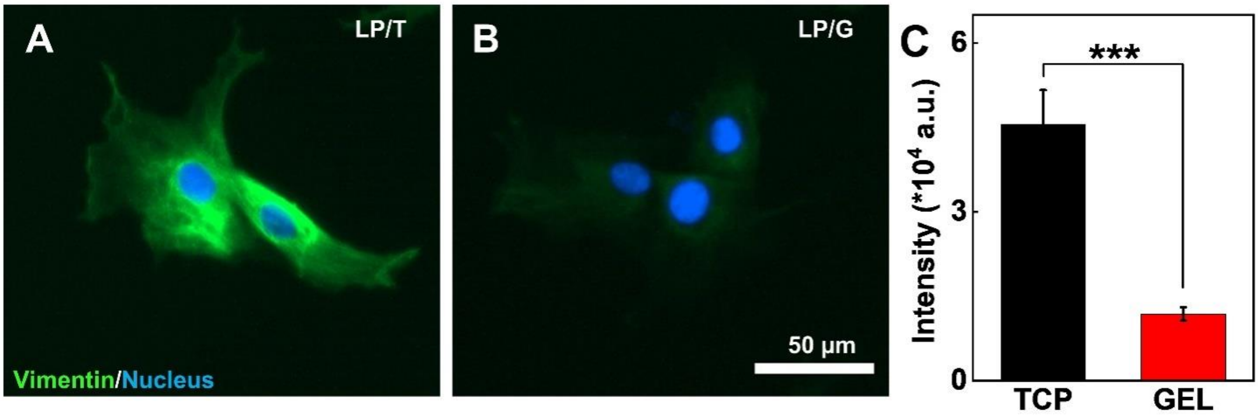

Figure S10: Effect of soft gel on overexpression of vimentin. Representative immunofluorescence images of LP UC-hMSCs from TCP (LP/T) (A) and from gel (LP/G) (B). Green shows vimentin and blue shows cell nucleus. (C) Quantification of vimentin intensity shows significant increase in LP cell from TCP compared to gel. Results are plotted as mean±s.e.m. Two tailed t-test ***p<0.0001 (N=3, n ≥ 50).
